# Design and Analysis of Self-Adapted Task Scheduling Strategies in Wireless Sensor Networks

**DOI:** 10.3390/s110706533

**Published:** 2011-06-27

**Authors:** Wenzhong Guo, Naixue Xiong, Han-Chieh Chao, Sajid Hussain, Guolong Chen

**Affiliations:** 1 College of Mathematics and Computer Science, Fuzhou University, Fujian 350108, China; E-Mails: fzugwz@163.com (W.G.); cgl@fzu.edu.cn (G.C.); 2 Department of Computer Science, Georgia State University, Atlanta, GA 30302, USA; 3 Institute of Computer Science & Information Engineering, National Ilan University, Taiwan; E-Mail: hcc@niu.edu.tw (H.-C.C.); 4 Computer Science, Fisk University, Nashville, TN 37208, USA; E-Mail: shussain@fisk.edu (S.H.)

**Keywords:** wireless sensor networks, task scheduling, particle swarm optimization, dynamic alliance

## Abstract

In a wireless sensor network (WSN), the usage of resources is usually highly related to the execution of tasks which consume a certain amount of computing and communication bandwidth. Parallel processing among sensors is a promising solution to provide the demanded computation capacity in WSNs. Task allocation and scheduling is a typical problem in the area of high performance computing. Although task allocation and scheduling in wired processor networks has been well studied in the past, their counterparts for WSNs remain largely unexplored. Existing traditional high performance computing solutions cannot be directly implemented in WSNs due to the limitations of WSNs such as limited resource availability and the shared communication medium. In this paper, a self-adapted task scheduling strategy for WSNs is presented. First, a multi-agent-based architecture for WSNs is proposed and a mathematical model of dynamic alliance is constructed for the task allocation problem. Then an effective discrete particle swarm optimization (PSO) algorithm for the dynamic alliance (DPSO-DA) with a well-designed particle position code and fitness function is proposed. A mutation operator which can effectively improve the algorithm’s ability of global search and population diversity is also introduced in this algorithm. Finally, the simulation results show that the proposed solution can achieve significant better performance than other algorithms.

## 1. Introduction

A wireless sensor network (WSN) is a system of spatially distributed sensor nodes that collect important information in the target environment. WSNs have been envisioned for a wide range of applications, such as battlefield intelligence, environmental tracking, and emergency response. Each sensor node has limited computation capacity, power supply and communication capability [1]. In a wireless sensor network (WSN), the usage of resources are usually highly related to the execution of tasks which consume a certain amount of computing and communication bandwidth. Parallel processing among sensors is a promising solution to provide the demanded computation capacity in WSNs, and task allocation and scheduling play an essential role in parallel processing [2]. Therefore, how to assign a task to its most appropriate sensor node and simultaneously balance the network load in the context of the uncertain and dynamic network environments represents an important and urgent issue in WSN studies.

As a typical problem of the area of high performance computing, task allocation and scheduling has been shown to be NP-complete. Several useful heuristic algorithms for task allocation and scheduling problems, such as MCT (Minimum Completion Time), Min-min (Min-min complete Time), Max-min (Max-min complete Time), Greed, Genetic Algorithm (GA) and so on, have been developed in the literature [3–7]. Due to the limited resource availability and shared communication medium, these existing algorithms cannot be directly implemented in WSNs. Thus, task allocation and scheduling have remained largely unexplored in WSNs until recently and we will summarize the existing work in Section 2.

In specific applications, the completion of tasks in WSNs is usually computation-intensive. With limited energy, computation and storage capacity, a WSN cannot complete its specific tasks without cooperative information exchange among several sensor nodes. For example, in a video sensor network application, the multimedia information is usually a computation-intensive task, which can usually be completed by the cooperation of several sensor nodes. Therefore distributed computation is important in WSNs. As a typical research field of the distributed artificial intelligence and the distributed computing, agent theories and technologies play an important role in modern computer science and applications. As some characteristics of WSNs are similar to those of multi-agent theories, such as, sensor nodes are capable of solving problems independently, and the WSN is distributed and self-organized, we can naturally attempt to apply multi-agent technologies to WSNs. Then, sensor nodes can be treated as agents that can create clusters independently by cooperating with each other to achieve their goals and coordinating their behaviors [8]. If a sensor node is regarded as an agent, the WSN is a kind of multi-agent system. However, the WSN is different from multi-agent system in some aspects, so multi-agent theories can’t be directly applied to WSNs.

The advantages of applying agent theories and technologies to the WSN are as follows:

1. Using agent and multi-agent system theory to model and simulate the WSN contributes to formally analyze and design network activities and organization.
2. Using agent software can expand the WSN intelligence and create an autonomous network system.
3. Using agent software can reduce the redundancy of sensing data and data flow.
4. Using agent software can save the energy of the WSN and effectively extend the network lifetime.
5. Using agent-based design theory and method can achieve the dynamic application of the WSN and highly flexible strategy of task scheduling.

As the WSN always works in an unknown dynamic environment, sensor nodes may fail in many cases, such as when they move or die from battery depletion. In this case, to extend the lifetime of the WSN, before they become disabled the remaining tasks of these nodes should be effectively transferred to other healthy nodes, which are able to finish these tasks. Thus, to solve this problem, this paper proposes a multi-agent-based self-adapted task scheduling strategy in WSNs. In this strategy, we first propose a dynamic alliance model for the task allocation problem with a view to prolong the lifetime, reduce the energy consumption and balance the network load. Then a discrete particle swarm optimization (PSO) for the dynamic alliance in our previous work [9], called DPSO-DA, is introduced in this paper. In the PSO-DA, we design a function considering the overall execution time of tasks, the energy consumption and the network balance. In addition, a mutation operator is introduced into DPSO-DA to maintain the population diversity and improve the global searching ability.

The rest of this paper is organized as following. In Section 2, we discuss related work. Section 3 describes the problem. In Section 4, we propose the algorithm for the dynamic alliance model of task allocation in WSNs. Section 5 introduces the multi-agent-based self-adapted task scheduling strategy. In Section 6, we present the simulation results. Section 7 gives the concluding remarks.

## 2. Related Work

As mentioned in the previous section, energy consumption is a fundamental challenge in WSNs due to their unique features. Most of those traditional solutions do not consider energy consumption during communication and task execution, so they cannot be implemented directly in WSNs. Thus, allocation and scheduling are topics that remain largely unexplored in WSNs. Recently, several algorithms have been proposed for the task allocation and scheduling problem. Giannecchini *et al*. proposed an online task scheduling mechanism called CoRAl [10] to allocate the network resources between the tasks of periodic applications in WSNs. However, CoRAl not only did not address mapping tasks to sensor nodes, but also failed to discuss explicitly energy consumption. An energy-constrained task mapping and scheduling called EcoMapS [2], which incorporates channel modeling, concurrent task mapping, communication and computation scheduling, and sensor failure handling algorithm, has also been presented. Tian *et al.* developed an application-independent task mapping and scheduling solution [11] in multi-hop WSNs, which not only provided real-time guarantees, but also implemented dynamic voltage scaling mechanism to further optimize energy consumption. Furthermore, a static energy-balanced task scheduling algorithm [12] was put forward, which assigned tasks with precedence constraints to a cluster of heterogeneous sensor nodes connected by a single-hop wireless network so as to maximize the lifetime of the sensor network. In addition, a novel task allocation strategy called Balanced Energy-Aware Task Allocation (BEATA) for collaborative applications running on heterogeneous networked embedded systems [7] was developed by Xie *et al*., and this strategy aimed at making the best trade-offs between energy savings and schedule lengths. Lin *et al.* advanced an adaptive energy-efficient multisensor scheduling for collaborative target tracking [13] in WSNs. Abdelhak *et al.* proposed EBSEL [14], an energy-balancing task scheduling and allocation heuristic whose main purpose is to extend the network’s lifetime, through energy balancing.

In those solutions, researchers mainly consider energy consumption during communication and task execution for task allocation and scheduling in WSNs. Some researchers develop multi-task scheduling algorithms for WSNs considering real-time and energy efficiency. However, due to some internal characteristics of WSNs, they have some disadvantages, such as dynamic network topology, limited energy, limited sensor node resources and unreliable sensing data, *etc*. The performances of task allocation and scheduling in WSNs should be improved in four aspects: real-time, economy, energy consumption and harmony. The PSO algorithm is a relatively recent swarm intelligence method developed by Kennedy and Eberhart [15]. The advantages of PSO over many other optimization algorithms are its simplicity of implementation and ability to converge to a reasonably good solution quickly. It has created a research hot spot and generated a massive volume of research results in only a few years [16–20] since the PSO algorithm was first proposed. A great number of experimental results show that PSO can solve nearly all kinds of optimization problems that can be solved by GA, so it is indeed a powerful and vital optimization tool. In our previous work [21], we proposed a discrete PSO algorithm called TO-PSO to solve the task allocation problem, which can get better results. However, it is easy to cause several machines sit idle when the number of tasks is small. This will reduce the balance of system load and increase additional consumption. As a result, the algorithm cannot provide reasonable scheduling solutions in a dynamic task number situation. In addition, the algorithm does not yet consider energy consumption during communication and task execution. In [22], we proposed a novel PSO algorithm to solve the task allocation problem. Later we found that dynamic alliance may have a fantastic performance. Therefore we applied dynamic alliance to the task allocation in WSNs in [23], which did not take self-adapted into account and was just compared with static alliance. In this paper, and inspired by multi-agent system theory, we first design a multi-agent model for WSNs. Then, in order to prolong the lifetime of the network, reduce the energy consumption and balance the network load, we propose a mathematical model of dynamic alliance for the task allocation problem and design a DPSO-DA algorithm with a well-designed particle position code and fitness function for this dynamic alliance model. Finally, we give an adaptive MAS-based task scheduling strategy, which self adaptively adjusts the status of unfinished tasks on the fault nodes in order to minimize the cost of the network recovery.

## 3. Problem Description

### 3.1. System Model

**Figure 1.**
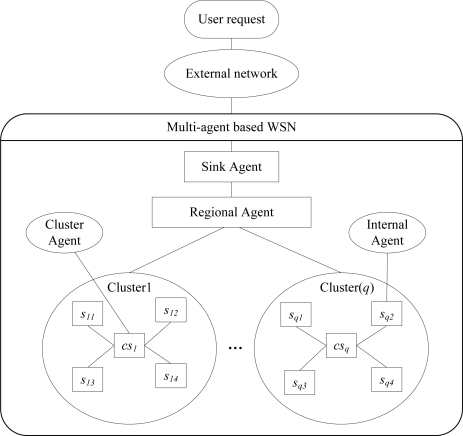
The multi-agent-based system architecture for WSNs.

As the hierarchical network topology has been widely used in WSNs, a multi-agent-based architecture for WSNs is proposed in this paper as shown in Figure 1. Due to the topological, spatial and deployment conditions, a WSN is always divided into several regions, each of which is divided into several clusters as well. Moreover, clusters may contain smaller clusters, for example, node 1 is the head of a 1st level cluster, which includes node 2 and node 3, and these two nodes are the heads of 2nd level clusters. Each cluster consists of a cluster agent (CA) and several member agents. In Figure 1, user requests are sent to the WSN through external networks, such as the Internet and satellites. The architecture is based on a three layer hierarchy of software agents. Generally, a user request is always transformed to an initial task, which is decomposable. Then, the initial task is decomposed into several smaller tasks with the same functionality. Acting as a high energy “gateway”, a sink agent is responsible for ensuring the interaction between the external network and the WSN. In addition, it also processes the final data obtained from the regional agents. At the regional layer, a regional agent manages a part of all the sensor agents, and performs local task allocation and data processing. Finally, a cluster agent collects the data from the agents in the cluster and performs some in-network operations, while simple agents usually implement some simple procedures, such as data sensing and local computing.

### 3.2. Task Allocation

Assume that a WSN is composed of *m* sensors and *n* independent tasks, where the tasks are competing for the sensors. The purpose of task allocation is to allocate the *n* tasks to the *m* sensors reasonably with shortest total execution time. We use a *n* × *m*-matrix called Execution Time Matrix (*ETM*) to express the tasks’ execution time on sensor nodes, and *etm_ij_* to express the execution time of task *i* on sensor *j*. So the *j*th node’s execution time of all tasks, which are allocated to this node, can be defined as follows:

(1) $$R(S_{j})=\sum_{i=1}^{n}{\mathrm{etm}}_{\mathrm{ij}}$$

Then, the system total execution time can be described as follows:

(2) $$K=\overset{m}{\underset{i=1}{\mathrm{Max}}}(R(S_{i}))$$

The total execution time *E* of all tasks implies the quality of the task allocation strategy. So lessening the value of *E* means that more tasks are assigned to the suitable sensors. It can be formulated as follows:

(3) $$E=\sum_{i=1}^{m}R(S_{i})$$

A good task allocation algorithm should guarantee not only the minimum *E*, but also the balance of network load. Load-balanced degree is a measure standard of the performance of WSN, and the load balance of WSN is better if load-balanced degree is bigger, we can define the balance of network load as follows:

(4) $$P=1-\sum_{i=1}^{m}\left(K-R(S_{i})\right)/(m\times K)\;P=1-\sum_{i=1}^{m}\left(K-R(S_{i}))/(m\times K\right)$$

In addition, when executing the tasks, sensor nodes must consume computing energy *C_local_*, and communicating energy *C_rou_*. Thus, the total energy consumption in WSNs is defined as follows:

(5) $$C=\sum_{i=1}^{m}\left(C_{\mathrm{local}}^{i}+C_{\mathrm{rou}}^{i}\right)$$

### 3.3. Dynamic Alliance Model

Due to these sensors’ different abilities of dealing with the tasks, an ideal distributed system should take the total execution time, the number of machines and the degree of cluster load into account. Dynamic alliances, known as “virtual enterprises”, are composed of a number of enterprises. These enterprises in the dynamic alliance use their respective advantages or core competencies in order to complete tasks efficiently [24]. Inspired by the idea of dynamic alliance, we transform the dynamic cluster alliance into the following multi-objective optimization problem [9]:

(6) $$\begin{array}{l} \mathrm{Minimize}\;(E)\\ R(S_{1})\approx R(S_{2})\approx \cdots \approx R(S_{m}),\text{that}\;\text{is},\;\mathrm{Maximize}\;(P)\\ \mathrm{Minimize}\;(C),\;\;\;\;\;\;C=\sum_{i=1}^{m}C_{\mathrm{local}}H_{i}+\sum_{i=1}^{m}\sum_{j=i+1}^{m}C_{\mathrm{rou}}H_{i}H_{j}\\ \mathrm{Minimize}(M),\;\;\;\;M=\sum_{i=1}^{m}H_{i},\;\;\text{where}\;\;H_{i}=\{\begin{array}{l} 1,\;\;if\;\;S_{i}\;\text{is}\;\text{selected}\\ 0,\;\;\mathrm{otherwise} \end{array}\\ s.t.\;1\leq M\leq m \end{array}$$

## 4. Algorithm for the Dynamic Alliance Model

### 4.1. Basic Particle Swarm Optimization

PSO is a population-based evolutionary algorithm which is initialized with a population of random solutions. In PSO, each particle is treated as a point with a velocity (*D*-dimensional vector) in a *D*-dimensional solution space. Each particle has a fitness value according to an objective function. Each particle adjusts its “flying” according to its own flying experience and its companions’ flying experience, and then closes to the minimum. The *i*th particle is represented as *X_i_* = (*X_i1_*, *X_i2_*, …, *X_iD_*). The velocity for the *i*th particle is represented as *V_i_* = (*V_i1_, V_i2_*, *…*, *V_iD_).* The best previous position (the position giving the best fitness value) of the *i*th particle is recorded and represented as *p_i_* = (*p_i1_*, *p_i2_*, …, *p_iD_*). The index of the best particle in the population is represented with the symbol *g*. At each step, the particles are manipulated according to the following equations:

(7) $$v_{\mathrm{id}}=w\times v_{\mathrm{id}}+c_{1}r_{1}(p_{\mathrm{id}}-x_{id})+c_{2}r_{2}(p_{\mathrm{gd}}-x_{\mathrm{id}})$$

(8) $$x_{\mathrm{id}}=x_{\mathrm{id}}+v_{\mathrm{id}}$$

where *w* is the inertia weight, *c_1_* and *c_2_* are two positive constants, called acceleration constants, *r_1_* and *r_2_* are two random numbers within the range [0, 1]. A constant, *V_max_* is often used to limit velocities of the particles and improve the resolutions of the search space.

According to Equations (7) and (8), the first part of Equation (7) represents the previous velocity, which provides the necessary momentum for particles to roam across the searching space. The second part, known as the “cognitive” component, represents the personal thinking of each particle. The cognitive component encourages the particles to move toward their own best positions found so far. The third part is known as “social” component, which represents the collaborative effect of the particles, during searching the global optimal solution.

### 4.2. Discrete Particle Swarm Optimization for the Dynamic Alliance Model

As the Equations (7) and (8) mentioned in the previous subsection, it is obvious that the basic PSO cannot be used to generate a discrete task allocation solution for its continuous nature, so some modification must be done to the original PSO. Since the PSO algorithm was proposed by Kennedy and Eberhart in 1995, many attempts have been made lately to apply the PSO algorithm to discrete combinatorial problems. Several discrete PSO algorithms have been put forth in the literatures, among which the discrete binary PSO algorithm [25], the discrete PSO algorithm for the traveling salesman problem [26], and the discrete PSO for the permutation flowshop sequencing problem with makespan criteria [27] have received the most attention. In this section, we introduce a DPSO-DA algorithm [9] to deal with the task allocation problem. In the proposed algorithm, a mutation operator is designed to maintain the population diversity and improve the global searching ability.

#### 4.2.1. Representation of Particles

In DPSO-DA algorithm, a particle’s position *X* denotes a dynamic alliance scheme. It can be represented as follows:

(9) $$X=(x_{1},x_{2},\cdots ,x_{i},\cdots ,x_{m}),\;\;\;1\leq i\leq m,x_{i}=\{0,1\}$$

where *m* is the number of sensors, the value of *x_i_* is the state whether the sensor is selected into dynamic alliance.

#### 4.2.2. Fitness Value Function

Inspired by the literature [28], we put forward an adaptive weight approach to construct the fitness value function. According to the two optimization objectives of the proposed dynamic alliance model, the total execution time *K* of task is lessened. Therefore, we can evaluate the value of *E* and *P* through computing the value of *K*. The fitness value function could be defined as follows:

(10) $$f(X)=\frac{K(X)}{K_{\text{max}}-K_{\text{min}}}+\frac{M(X)}{M_{\text{max}}-M_{\text{min}}}+\frac{C(X)}{C_{\text{max}}-C_{\text{min}}}$$

where *K_max_* and *K_min_* represent the maximum and minimum value of *K* respectively, *M_max_* and *M_min_* are the maximum and minimum member number of the dynamic alliance respectively, *C_max_* and *C_min_* are the maximum and minimum value of energy consumption *C* respectively, *K(X)* is the *K* value of the particle *X*, *M(X)* is the member number of the dynamic alliance, and *C(X)* is the *C* value of the particle *X.*

Here, we adopt the heuristic method based on the maximum time span, similar to the literature [29], to calculate the value of *K*. Here, we first compute the time span of the tasks which have not been dealt with, then allocate the tasks with the maximum time span to the processor with the minimum execution. This method can obtain good results in that it assigns the tasks according to the forecast information.

#### 4.2.3. Basic Operator

The velocity *V* of the particle represents the changed value of this particle’s position and it can be described as follows:

(11) $$V=(v_{1},v_{2},\cdots ,v_{i},\cdots ,v_{m}),\;\;1\leq i\leq m,v_{i}=\{0,1,2\}$$

where *m* is the number of the sensors.

In Equation (11), if *v_i_* equals 2, it implies that the state of the *i*th sensor is not changed; otherwise, the state of this sensor equals *v_i_*. Since the task allocation is a discrete problem, the operators in standard PSO should be redefined to solve this problem.

**Definition 1 (Subtraction Operator −)** Suppose *X_i_* and *X_j_* are the positions of the *i*th and *j*th particle respectively, then *V* = *X_i_* – *X_j_* expresses the change of position and each dimension’s value of *V* can be formulated as follows:

(12) $$v_{k}=\{\begin{array}{l} 2,\;\;\mathrm{if}\;x_{i,k}=x_{j,k}\\ x_{j,k},\;\;\mathrm{else} \end{array}$$

**Definition 2 (Additive Operator +)** Suppose *X_i_* is the position of the *i*th particle*,* then the particle’s position can be updated by the effect of velocity *V*, that is, $X_{i}^{\prime }=X_{i}+V$. And each dimension’s value of the new position $X_{i}^{\prime }$ can be formulated as follows:

(13) $$x_{\mathrm{ij}}^{\prime }=\{\begin{array}{l} x_{\mathrm{ij}}\;\;\;if\;v_{j}=2\\ v_{j}\;\;\;\mathrm{otherwise} \end{array}$$

**Definition 3 (Multiplication Operator ×)** Suppose *V_i_* is the velocity of the *i*th particle, then the particle’s speed can be updated by the follows:

(14) $$V_{i}^{\prime }=c_{1}\times V_{i}\times c_{2}$$

where 1≤*c*_1_≤*c*_2_≤‖*V_i_*‖ and ‖*V_i_*‖ is the dimension of *V_i_*.

And each dimension’s value of the new speed $V_{i}^{\prime }$ is defined as follows:

(15) $$v_{\mathrm{ij}}^{\prime }=\{\begin{array}{l} v_{\mathrm{ij}},\;\;if\;j\in [c_{1},c_{2})\\ 2,\;\;\;\mathrm{else} \end{array}$$

Based on the basic operator mentioned above and considering the disadvantages of the mutual interference in discrete PSO, the particles can be manipulated according to the following Equations:

(16) $$\begin{array}{l} X=X+c_{1}\times (P_{b}-X)\times c_{2},1\leq c_{1}\leq c_{2}\leq ||X||\\ X=X+c_{3}\times (P_{g}-X)\times c_{4},1\leq c_{3}\leq c_{4}\leq ||X|| \end{array}$$

where *P_b_* and *P_g_* represent the particle’s history best value and the global best value respectively, and ‖*X*‖ is the dimension of *X*.

#### 4.2.4. Mutation Operator

**Definition 4 (Particle Similarity)** Particle similarity *S_ij_* expresses similarity between the particle *i* and *j*, that is, the proportion of the same genes between particle *i* and *j* to the total number of genes. It can be described as follows:

(17) $$S_{ij}=\frac{1}{m}\sum_{k=1}^{m}\left(i_{k}=j_{k}?\;\;\;1:0\right)$$

where *k* is the *k*-th gene, and *m* is the number of genes.

**Definition 5 (Particle Diversity)** Particle diversity *Q_i_(t)* is based on the similarity among the particle *i*, its history best value and the global best value. It can be described as follows:

(18) $$Q_{i}(t)=1-\frac{1}{3}(S_{i,Pb}(t)+S_{i,Pg}(t)+S_{Pb,Pg}(t))$$

where *P_b_* and *P_g_* represent the particle’s history best value and the global best value respectively.

Then we can compute the population diversity as follows:

(19) $$D=1-\frac{1}{m(m-1)/2}\sum_{i=1}^{m}\sum_{j\neq i}^{m}S_{ij}$$

**Definition 6 (Consolidation Operator ⊕)** Suppose *X_1_* is the particle’s current position and *X_2_* is the objective position, then the particle’s velocity *pV* can be obtained with the effect of consolidation operator, that is, *pV* = *X*_2_ ⊕ *X*_1_. Each dimension’s value of the velocity *pV* can be formulated as follows:

(20) $$pv_{i}=\{\begin{array}{l} x_{1,i},\;\;\;if\;x_{1,i}\neq x_{2,i}\\ 1-x_{1,i},\;\;\;else \end{array}$$

During the iterative process of the standard PSO, population diversity is reduced and the ability of global exploration is restricted because the particles converge to the global best value gradually. To avoid falling into the local optimum, enhance the population diversity and improve the ability of global exploration, an improved method by monitoring the particle diversity *Q_i_(t)* and the population diversity *D* is adopted in the proposed DPSO-DA. In the method, it will execute the mutation operator on the all particles to guarantee the population diversity when the population diversity *D* is less than the threshold *D_0_*, and will execute the mutation operator on the particle *i* to guarantee the particle diversity and escape from the best value when the particle diversity *Q_i_(t)* is less than the threshold.

#### 4.2.5. Algorithm Overview

As the components of the DPSO-DA algorithm [9] mentioned in previous subsections, then the details of this algorithm will be described in this section (see Algorithm 1):

**Algorithm 1.** The Discrete PSO Algorithm for DA Model of Task Allocation (DPSO-DA).

**Step 1:** Initialize population; **Step 2:** Calculate the fitness value; **Step 3:** Update the particles’ position according to Equation (16); **Step 4:** Update the local best value; **Step 5:** Update the global best value; **Step 6:** For each particle, execute the mutation operator on this particle if its diversity is less than the threshold; **Step 7:** Execute the mutation operator on the all particles if the population diversity *D* is less than the threshold *D_0_*; **Step 8:** If the termination conditions are satisfied, then the algorithm terminates, otherwise go to **Step 2.**

#### 4.2.6. Complexity Analysis

**Lemma 1** Assume the dimension of the particle is *M*, the population size is *S*, the maximum number of iterations is *I*, and the size of the execution time matrix is *T*, then the time complexity of DPSO-DA is *O(I* × *S* × *T* × *M)*.

**Proof:** In the DPSO-DA algorithm, the time complexity of initializing swarm, updating the particles’ position, updating the local best value and updating the global best value are all *O(S* × *M).* The time of calculating the fitness value of the all particles is *O(M* × *S* × *T)*, the time of calculating the particles’ diversity is *O(M* × 3*)*, and the time of calculating the population diversity is *O(S* × *M).* The complexity of the mutation operator in step 6 and step 7 are *O(M* × 2*)* and *O(S* × *M)* respectively. So the time complexity of this algorithm is *O(I* × *(S* × *M + M* × *S* × *T + S* × *M + S* × *M + M* × 3 *+ M* × 2 + *S* × *M + S* × *M))*, that is *O(I* × *S* × *T* × *M).*

## 5. Multi-Agent-Based Self-Adapted Tasks Scheduling Strategy

**Figure 2.**
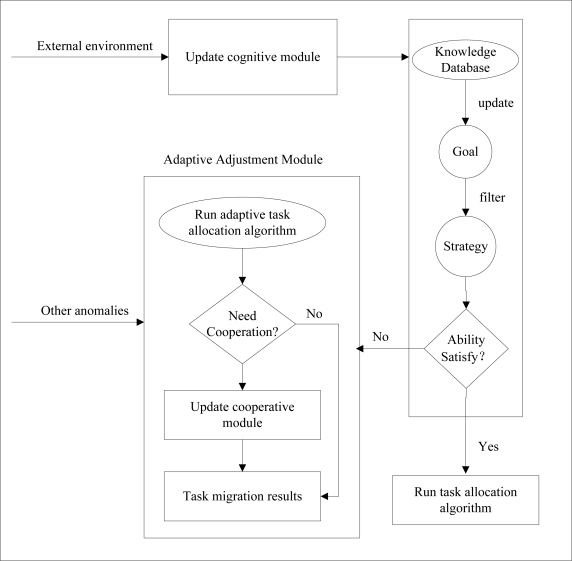
The multi-agent-based self-adapted task scheduling model in WSNs.

Sensor nodes use limited, generally irreplaceable, power sources and WSNs always work in dynamic environments where the network topology rapidly changes and connections are instable. Thus, when the sensor nodes fail, as a results of moving or dying as a result of battery depletion, these sensor agents should be able to sense their implementation environment and react autonomously to the changes by moving the unexecuted tasks to their neighbors and adaptively readjusting the network topology. Recently, task allocation has become an important and urgent issue in WSNs, and energy efficiency is a key concern in WSNs, so energy-efficient task allocation should be taken into account. However most of current works focus on the static network environment in WSNs. Although people have done some research on dynamic networks, they just simply move the unexecuted tasks to their healthy neighbors. In this paper, combining the cognitive module and the adaptive adjustment module, we propose a multi-agent-based adaptive task assignment model for WSNs, as shown in Figure 2. Once an agent senses the changes of external environment, the agent updates the knowledge database and the goal of the cognitive module. Then, if the agent satisfies the requirements, such as, energy requirement, communication and computation ability, it will select a proper scheme to allocate the given tasks. Otherwise it should run adaptive adjustment module. Moreover, for other exceptions of the agents, such as load-imbalance, the network should use the adaptive adjustment module of the agents to dynamically adjust itself.

WSN is a kind of network with a large number of nodes and limited resources, which always adopts a multi-hop route mechanism. As sensor nodes are distributed in a large region, we always divide them into many partitions to manage, and several blocks are managed by a management node (MA). In order to save energy consumption, sensor agents only conserve the information of their neighbor agents. Thus, for the sake of energy saving and easy management, a MA conserves the information of all agents. So, when an agent wants to acquire its non-neighbor agent’s information, it can interact with its MA.

Here, we define two vectors conserved in MAs, load vector and remainder energy vector represent current load and current remainder energy of agents in a region respectively. They are defined as follows:

(21) $$L=\{L_{1},L_{2},\cdots L_{k}\}$$

(22) $$\mathrm{ER}=\{{\mathrm{ER}}_{1},{\mathrm{ER}}_{2},\cdots {\mathrm{ER}}_{k}\}$$

**Figure 3.**
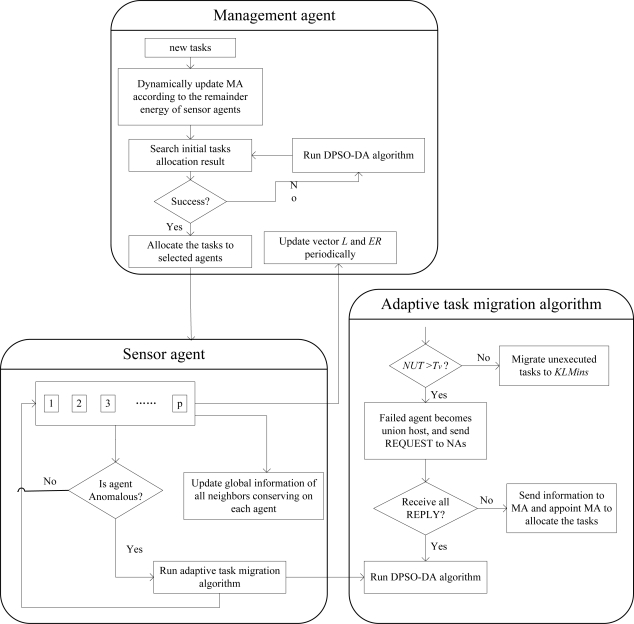
The self-adapted task allocation strategy.

Each agent can sense its load and remainder energy. When detecting that its load is more than the threshold *L_0_* or its remainder energy is below the threshold *ER_0_*, the agent will run an adaptive adjustment algorithm, and inform its neighbor agents (NAs) and MA. In addition, defining a threshold *T_v_*, when unexecuted tasks of failed agent is more than *T_v_*, it will run task allocation algorithm, or move those tasks directly to several agents with minimum load. Thus, a self-adapted task allocation strategy with dynamic feedback to the adaptive adjustment algorithm module in Figure 2 is proposed as shown in Figure 3.

Figure 3 is composed of two modules which are function module of management agents (MAs) and sensor agents (SA). As shown in Figure 3, when the load of an agent is more than the threshold, or the agent is in high-load continuously, or the remainder energy is below the threshold, the agent must effectively migrates its unexecuted tasks to other health agents before it fails to ensure the performance of the whole network. In addition, in Figure 3, *NUT* represents the number of unexecuted tasks, and *KLMins* denotes as the *k* smallest load agents.

## 6. Simulation Results and Analysis

### 6.1. Test Data

Similar to the method of generating data in [6], the method includes three parameters: task heterogeneity *ϕ_d_*, sensor heterogeneity *ϕ_m_*, and data consistency. The details of generating data can be seen in [6]. If task heterogeneity is low, then *ϕ_d_* equals 100, which means that the difference among tasks is small, otherwise *ϕ_d_* equals 3,000. Similarly, if the sensor heterogeneity is low, then *ϕ_m_* equals 10, which means that the difference among sensors is small, otherwise *ϕ_m_* equals 1,000. Firstly, we generate a *n*-vector *B* randomly, and each element *b_i_*∈ [1,*ϕ_d_* − 1], then generate a *n* × *m*-matrix *E* randomly too, and each element *x_ij_*∈ [1,*ϕ_m_* − 1], finally calculate estimated execution time, *etm*_ij_ = *b*_i_ × *x*_ij_, *etm*_ij_∈ [1,*ϕ_d_* × *ϕ_m_* − 1]. Considering data consistency, if consistent, then sort elements of each row in *ETM*, which make sure that for elements in any row, if *k* < *l* then *etm*_ik_ < *etm*_il_; if semi-consistent, then sort elements of even columns in every row separately, which make sure that for any element in even columns of any row, if *k* < *l* then *etm*_ik_ < *etm*_il_; if inconsistent, then do nothing with *ETM*.

Here we name the test data according to this format: *x*-*y*-*z*. In this format, *x* is data streams heterogeneity, while taking ‘*l*’ means low (*ϕ_d_* equals 100), and ‘*h*’ means high (*ϕ_d_* equals 3,000); *y* is machines heterogeneity, similarly to *x*, ‘*l*’ means low (*ϕ_m_* equals 10), and ‘*h*’ means high (*ϕ_m_* equals 1,000), and *z* is data consistency, ‘*c*’ means consistent, ‘*s*’ means semi-consistent and ‘*d*’ means inconsistent.

### 6.2. Results and Analysis

Let us consider a task allocation problem with 128 tasks and 16 sensor nodes, the internal energy consumption of sensor nodes is the consumption cost of executing 100 estimated runtimes, which is between 0.2 and 1.5 s with a normal distribution whose center point is 1, while communication consumption of WSN is represented as a *m* × *m* matrix which is between 3 and 7 with a normal distribution whose center point is 5. We assume that every task can be equally accomplished by any sensor, regardless of its position. Then we perform the DPSO-DA algorithm with each test data for five times and take the best solution. After a lot of tries, DPSO-DA can get satisfied solution in short time (10 s level) when parameters are set in this way: the number of iterations is 1,000, the population size is 100, the threshold of particle diversity is 0.2, and the threshold of population diversity is 0.4.

**Figure 4.**
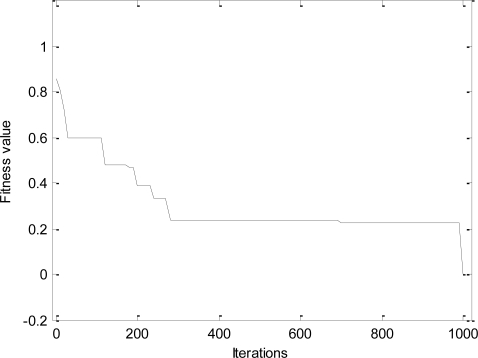
Convergence process.

**Figure 5.**
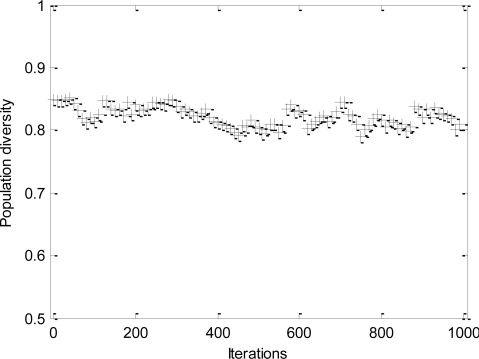
Change of population diversity.

The convergence process and the change of population diversity for *l-h-c* problem are shown, respectively, in Figures 4 and 5. The results imply that the proposed DPSO-DA algorithm has a good convergence and its population diversity also keeps a high level all the time. In addition, the DPSO-DA algorithm for another problem can also get similar performance as with the *l-h-c* problem.

Then we perform DPSO-DA algorithm for all the following possible problems, and obtain the results as showed in Table 1. From Table 1, we can know that the DPSO-DA algorithm can choose the appropriate agents to constitute the cluster alliance with a high level of load balance because the values of load balance in all problem types are all better than 0.95.

**Figure 6.**
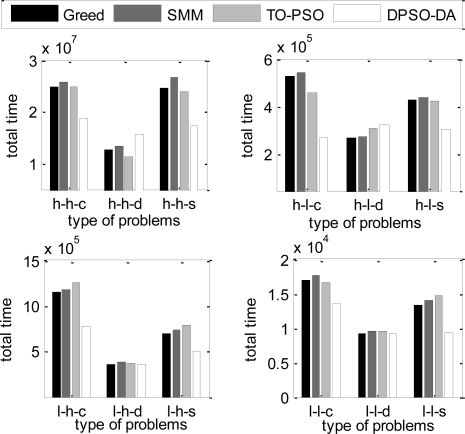
The total execution time under different task allocation solutions.

**Figure 7.**
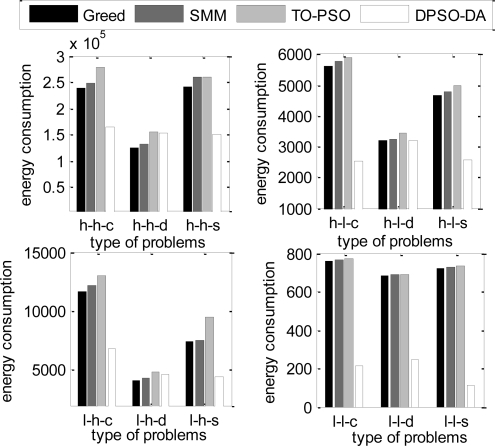
The total energy consumption under different task allocation solutions.

**Figure 8.**
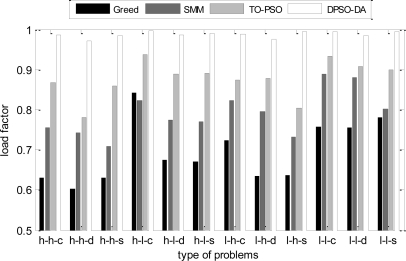
The network load factors under different task allocation solutions.

In the next simulation, the total execution time, the energy consumption and the degree of load balance calculated using DPSO-DA, TO-PSO [21], Greed [4] and SMM [6] are shown in Figures 6, 7 and 8, respectively.

From Figures 6 and 7, we can see that the DPSO-DA algorithm can get results which are better than Greed, SMM and TO-PSO in most problems, and its allocation scheme can not only reduce energy consumption but also ensure the minimum total completion time. From Figure 8, we can see that the DPSO-DA algorithm, which outperforms Greed, SMM and TO-PSO in the all problems, can also get a good load balance which is better than that of Greed, SMM and TO-PSO. This is because the load balance is considered in our proposed DPSO-DA algorithm. Therefore, to ensure the minimum total execution time and the load balance, the DPSO-DA algorithm should select appropriate machines to constitute a cluster alliance according to different task sets.

**Figure 9.**
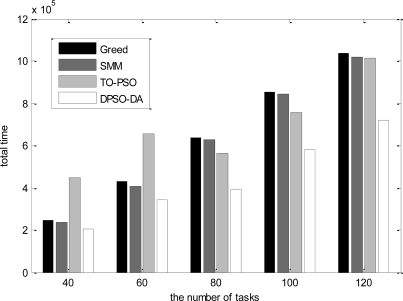
The total execution time for the dynamic tasks under different task allocation solutions.

**Figure 10.**
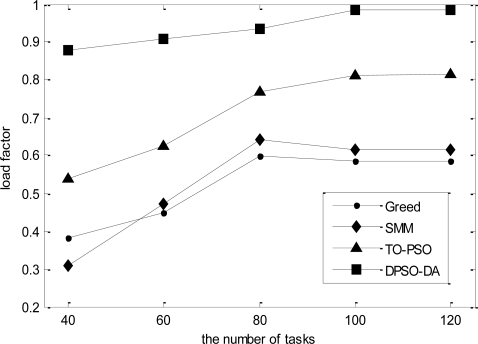
The network load for the dynamic tasks under different task allocation solutions.

**Figure 11.**
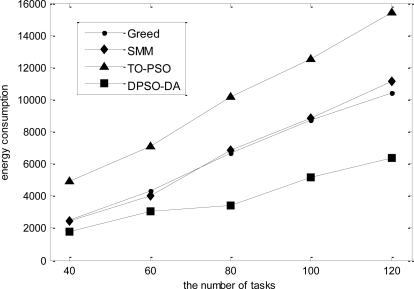
The total energy consumption for the dynamic tasks under different task allocation solutions.

In the third simulation, we consider 16 sensor nodes and vary the number of tasks from 40 to 120. The total execution time, the degree of load balance and the total energy consumption calculated for *l-h-s* problems using DPSO-DA, TO-PSO, Greed and SMM are shown in Figures 9, 10 and 11, respectively.

As shown in Figures 9–11, we know that Greed and SMM will let some sensors remain idle and cause a low network load if the number of tasks is small. Although the performance of the total execution time is acceptable, the degree of load balance cannot be improved with an increased number of tasks, while will be reduced when the size of tasks becomes larger than 80. The total execution time of TO-PSO is a little smaller than that of Greed and SMM, but it can achieve better load performance than Greed and SMM. In addition, the degree of load balance cannot be reduced with the increased number of tasks. From Figures 9–11, we also can see that DPSO-DA outperforms TO-PSO in the performance of total execution time and load balance with the increased number of tasks.

**Figure 12.**
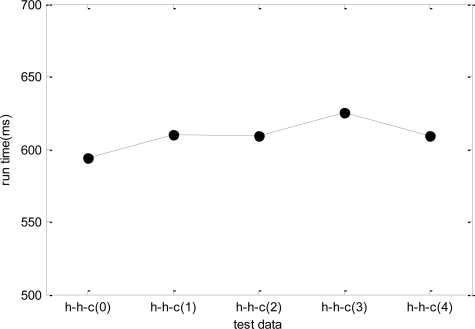
The runtime of DPSO-DA for five groups of *h-h-c* data.

**Figure 13.**
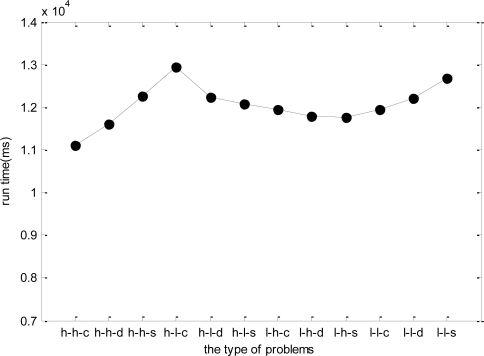
The DPSO-DA runtime for different types of test data.

In the last simulation, due to the dynamic change nature of WSNs, the test data may be different even for the same problem. Therefore, the stability of DPSO-DA algorithm needs to be considered when the test data changes. Taking *h-h-c* for example, we generate five groups of test data of *h-h-c* to test the DPSO-DA algorithm, and the results are shown in Figure 12. As WSN is a kind of network system with strong real-time, the DPSO-DA runtime has a great influence on the performance of any adaptive task scheduling strategy, that is, if the runtime of the algorithm is too long, *ETM* will not be renewed in time and the allocation scheme will not be optimal which may influence the results. In addition, as WSN is application-oriented, different applications have different network configuration, property value and so on. Therefore, we test for 12 types of problems respectively, and obtain their runtime in Figure 13.

From Figure 12, we know that the DPSO-DA runtime is relatively stable for the same type of problem. From Figure 13, for different problems, the DPSO-DA algorithm runtime has little deviation generally. Thus, we can set a proper updating time gap for *ETM* to ensure that management platform has enough time to renew the task allocation scheme. To do this, the total time cost of adaptive task adjustment is in the range of an affordable system.

## 7. Conclusions and Future Work

Due to several characteristics of wireless sensor networks, this paper develops a self-adapted task scheduling strategy inspired by the multi-agent system theory. In the proposed strategy, we build a dynamic alliance model for task allocation in WSNs and propose a DPSO-DA algorithm with a well-designed particle position code and fitness function for this dynamic alliance model. In the DPSO-DA algorithm, we design a function taking into account the overall execution time of tasks, the energy consumption and the network balance and incorporate a mutation operator to maintain the population diversity and improve the global searching ability. Simulation results show that the proposed strategy achieves a good balance between local solutions and global exploration, effectively reduces the computation time of network and the network energy consumption, and balances the whole network load. In future work, we will consider the real multi-objective optimization of this problem and extend our current work to actual scenarios.

## Figures and Tables

**Table 1.** The result of DPSO-DA algorithm

**Type**	**Dynamic alliance structures**	**The load balance**	**The number of nodes**
*h-h-c*	1, 2, 3, 5, 12	0.986548	5
*h-h-d*	1, 2, 6, 8, 9, 10, 12, 13, 16	0.970726	9
*h-h-s*	1, 2, 3, 4, 8, 12, 16	0.984089	7
*h-l-c*	1, 2, 3, 4	0.996335	4
*h-l-d*	2, 5, 6, 7, 10, 13, 15, 16	0.985648	8
*h-l-s*	1, 2, 3, 4, 10	0.989632	5
*l-h-c*	1, 2, 3, 4, 9	0.98794	5
*l-h-d*	4, 6, 7, 8, 9, 10, 12, 13, 14, 15, 16	0.948361	11
*l-h-s*	1, 2, 3, 8	0.993726	4
*l-l-c*	1, 2, 3, 8, 16	0.993997	5
*l-l-d*	6, 7, 8, 11, 12, 15	0.980782	6
*l-l-s*	1, 2, 4, 6	0.994178	4
